# The Acute Effects of 5 Fluorouracil on Skeletal Muscle Resident and Infiltrating Immune Cells in Mice

**DOI:** 10.3389/fphys.2020.593468

**Published:** 2020-12-07

**Authors:** Brandon N. VanderVeen, Alexander T. Sougiannis, Kandy T. Velazquez, James A. Carson, Daping Fan, E. Angela Murphy

**Affiliations:** ^1^Department of Pathology, Microbiology, and Immunology, University of South Carolina School of Medicine, Columbia, SC, United States; ^2^AcePre, LLC, Columbia, SC, United States; ^3^Department of Physical Therapy, College of Health Professionals, University of Tennessee Health Science Center, Memphis, TN, United States; ^4^Department of Cell Biology and Anatomy, University of South Carolina School of Medicine, Columbia, SC, United States

**Keywords:** chemotherapy, monocytes, macrophages, skeletal muscle, bone marrow

## Abstract

5 fluorouracil (5FU) has been a first-choice chemotherapy drug for several cancer types (e.g., colon, breast, head, and neck); however, its efficacy is diminished by patient acquired resistance and pervasive side effects. Leukopenia is a hallmark of 5FU; however, the impact of 5FU-induced leukopenia on healthy tissue is only becoming unearthed. Recently, skeletal muscle has been shown to be impacted by 5FU in clinical and preclinical settings and weakness and fatigue remain among the most consistent complaints in cancer patients undergoing chemotherapy. Monocytes, or more specifically macrophages, are the predominate immune cell in skeletal muscle which regulate turnover and homeostasis through removal of damaged or old materials as well as coordinate skeletal muscle repair and remodeling. Whether 5FU-induced leukopenia extends beyond circulation to impact resident and infiltrating skeletal muscle immune cells has not been examined. The purpose of the study was to examine the acute effects of 5FU on resident and infiltrating skeletal muscle monocytes and inflammatory mediators. Male C57BL/6 mice were given a physiologically translatable dose (35 mg/kg) of 5FU, or PBS, i.p. once daily for 5 days to recapitulate 1 dosing cycle. Our results demonstrate that 5FU reduced circulating leukocytes, erythrocytes, and thrombocytes while inducing significant body weight loss (>5%). Flow cytometry analysis of the skeletal muscle indicated a reduction in total CD45+ immune cells with a corresponding decrease in total CD45+CD11b+ monocytes. There was a strong relationship between circulating leukocytes and skeletal muscle CD45+ immune cells. Skeletal muscle Ly6c^High^ activated monocytes and M1-like macrophages were reduced with 5FU treatment while total M2-like CD206+CD11c- macrophages were unchanged. Interestingly, 5FU reduced bone marrow CD45+ immune cells and CD45+CD11b+ monocytes. Our results demonstrate that 5FU induced body weight loss and decreased skeletal muscle CD45+ immune cells in association with a reduction in infiltrating Ly6c^High^ monocytes. Interestingly, the loss of skeletal muscle immune cells occurred with bone marrow cell cycle arrest. Together our results highlight that skeletal muscle is sensitive to 5FU’s off-target effects which disrupts both circulating and skeletal muscle immune cells.

## Background

The increase in 5-year survival rate among cancer patients has increased focus on quality of life to improve patient outcomes (Curt et al., 2000). In addition to cancer-associated wasting and functional decrements, the most commonly prescribed chemotherapies have pervasive off-target effects that have been reported to impact quality of life (Iacovelli et al., 2014; Lee et al., 2014; Polk et al., 2014; Ribeiro et al., 2016). 5 fluorouracil (5FU) has been the first-choice chemotherapy drug for several cancer types for many years (van Kuilenburg and Maring, 2013; Giuliani and Bonetti, 2016; Lee et al., 2016; McQuade et al., 2017); however, 5FU negatively impacts the gastro-intestinal system (Lee et al., 2014; Ribeiro et al., 2016; Sougiannis et al., 2019), cardiovascular system (Polk et al., 2014; Phillips et al., 2018), hematopoietic system (Kvinnsland, 1999; Shitara et al., 2009; Han et al., 2012), and has recently been shown to directly disrupt skeletal muscle (Barreto et al., 2016b; Botsen et al., 2018; Williams et al., 2018). Disruptions to skeletal muscle homeostasis contributes to functional dependency and poor treatment outcomes and ultimately leads to increased healthcare costs and decreased survival (Barreto et al., 2016b). Currently, there are no Food and Drug Administration approved therapies for chemotherapy-induced cachexia despite the importance of skeletal muscle and lean mass in sustaining 5FU’s therapeutic efficacy and patient quality of life (Sandini et al., 2018; Williams et al., 2018). This is not entirely surprising given that very little is known about the mechanisms responsible for 5FU-induced skeletal muscle dysfunction. Thus, identifying the factors driving chemotherapy-induced skeletal muscle dysfunction is critical to developing effective interventional therapies.

Despite 5FU-induced leukopenia remaining a hallmark of treatment (Shitara et al., 2011; Sougiannis et al., 2019), investigations into the impact of 5FU on skeletal muscle have largely focused on metabolism (Barreto et al., 2016a, b). Notably, there is a dearth of evidence on the influence of 5FU on skeletal muscle inflammation – a process that is known to play a role in skeletal muscle homeostasis (Costamagna et al., 2015). Indeed, inflammation can play a paradoxical role in skeletal muscle homeostasis. During normal conditions pro-inflammatory cytokines are required to balance anabolism and catabolism and to maintain normal myogenic processes. However, during disease conditions, pro-inflammatory cytokines can induce catabolic pathways that impair skeletal muscle integrity and function (Sharma and Dabur, 2020). To date, our understanding of 5FU-induced inflammatory changes is limited to circulating inflammatory cytokines and intrinsic inflammatory signaling. Additionally, the available studies highlight equivocal results showing increased circulating interleukin (IL) 6, tumor necrosis factor α (TNFα), monocyte chemoattractant protein (MCP) 1 (Wang et al., 2012; Mahoney et al., 2013, 2014), with reduced or unchanged skeletal muscle inflammatory protein expression (Barreto et al., 2016a, b). Given the importance of skeletal muscle to quality of life in chemotherapy patients along with the well-documented effects of inflammation on skeletal muscle homeostasis, it is important to assess inflammatory mediators as a potential target for chemotherapy-induced skeletal muscle dysfunction (Kvinnsland, 1999; Yamanaka et al., 2007; Shitara et al., 2009; Baechler et al., 2010; Han et al., 2012; Abraham et al., 2015).

The lack of evidence on 5FU associated perturbations in skeletal muscle inflammation is consistent with a scarcity of literature on 5FU effects on skeletal muscle immune cells. Monocytes, or more specifically macrophages, are the most abundant skeletal muscle immune cell which function to regulate tissue turnover and homeostasis (Tidball, 2017). Targeting macrophages is emerging as a potential key regulator of chemotherapeutic efficacy given the importance of tumor associated macrophages (TAM) in tumorigenesis, tumor vascularization, and local immunosuppression (Mantovani and Allavena, 2015); however, the effects of 5FU on skeletal muscle macrophages is largely unexplored. Resident skeletal muscle monocytes are classically characterized as CD11b+Ly6c^Low^ monocytes and F4/80+CD11c-CD206- (quiescent – M0) macrophages (Krippendorf and Riley, 1993; Tidball, 2017). Circulating CD11b+Ly6C^High^ activated monocytes, recruited by MCP-1, extravasate the muscle (Deshmane et al., 2009; Liao et al., 2018) and either remain CD11b+Ly6C^High^ or differentiate to F4/80+CD11c+CD206- pro-inflammatory, pro-phagocytic (M1-like) macrophages which secrete pro-inflammatory cytokines, IL-6, IL-1β, TNFα, and interferon (IFN) γ (Frenette et al., 2003; Guilliams et al., 2014). These M1-like macrophages will then down regulate CD11c expression and increase CD206+ to reflect a more anti-inflammatory, pro-fibrotic (M2-like) macrophage which secrete anti-inflammatory cytokine IL-10 and pro-fibrotic cytokine transforming growth factor (TGF) β (Gordon and Taylor, 2005; Arnold et al., 2007; Reidy et al., 2019). Proper balance of these immune cell phenotypes and maintenance of immune cell number are vital for skeletal muscle homeostasis. Thus, determination of 5FU’s effects on skeletal muscle immune populations is essential for the development of effective treatment strategies.

Skeletal muscle immune cell depletion has been demonstrated to delay recovery and disrupt extracellular matrix remodeling leading to fibrosis, weakness, and metabolic homeostatic imbalance (Farini et al., 2007; Liu et al., 2017). While results pertaining to intrinsic skeletal muscle inflammatory signaling with several chemotherapies are equivocal, leukopenia has been well established (Kvinnsland, 1999; Yamanaka et al., 2007; Shitara et al., 2009; Baechler et al., 2010; Han et al., 2012; Abraham et al., 2015). The overall purpose of the current study was to investigate the acute effects of 5FU on resident and infiltrating skeletal muscle monocytes and inflammatory mediators. We hypothesized that an acute dosing regimen of 5FU would deplete circulating and skeletal muscle monocytes and reduce associated inflammatory cytokines consistent with systemic leukopenia. Our results demonstrate that 1 cycle of 5FU was sufficient to induce significant body weight loss and leukopenia associated with a loss of total skeletal muscle immune cells and a reduction in select inflammatory mediators. Additionally, we show 5FU induced bone marrow cell cycle arrest which is likely to contribute to the observed loss of infiltrating skeletal muscle monocytes.

## Materials and Methods

### Animals

Eighteen male C57BL/6 mice were purchased from Jackson Laboratories at 4 weeks of age and housed in the Department of Laboratory Animal Resources at the University of South Carolina. Mice were either group housed (*n* = 12) or singly housed to measure food intake (*n* = 6) and kept on a 12:12-h light-dark cycle. Animals were placed on a purified AIN-76A (Bio-Serv, Frenchtown, NJ, United States; catalog#:F1515) diet for 5 weeks prior to any experimental procedures. Body weights were measured weekly, and animals were monitored for signs of distress. Animals were given food and water *ad libitum* throughout the duration of the study. All animals were fasted 5 h prior to tissue collection. Mice were anesthetized with isoflurane and hindlimb muscles, select organs, and both femurs were carefully dissected, weighed, and either snap frozen in liquid nitrogen or placed in the appropriate buffers for flow cytometry analysis. All animal experiments were approved by the University of South Carolina’s Institutional Animal Care and Use Committee.

### Experimental Design

At 14 weeks of age mice were randomized into two groups, Control (*n* = 9) and 5FU (*n* = 9). 5FU was solubilized in PBS at 3.5 mg/mL and administered to the mice at 35 mg/kg i.p. once daily for 5 days. This dosing regimen has been previously shown to be comparable to clinical doses and recapitulates 1 cycle of chemotherapy (Phillips et al., 2018; Sougiannis et al., 2019). Control mice received a PBS injection. Tissue was collected and the animals were euthanized 24 h following the final injection.

### Blood Analysis

Blood was collected at euthanasia via the inferior vena cava, placed in an EDTA coated vacutainer (VWR, Suwanee, GA, United States; catalog#:454428) and stored briefly on ice until analysis. A complete blood count was performed using the VetScan HMT (Abaxis, Union City, CA, United States) for determination of white blood cells (WBCs), lymphocytes (LYM), monocytes (MON), neutrophils (NEU), red blood cells (RBCs), Hemoglobin (HGB), Hematocrit (HCT), and platelets (PLT).

### Flow Cytometry

Both quadriceps were excised, minced in Dulbecco’s Modified Eagle Medium (DMEM), and cells were extracted using the skeletal muscle dissociation kit (Miltenyi Biotec, Auburn, CA, United States; cat#; 130-098-305) following the manufacture’s instruction. Both quadriceps were pooled to obtain a sufficient number of cells for each analysis without pooling animals (*n* = 9/group). Skeletal muscle cells were suspended in flow buffer (0.5% BSA, 2 mM EDTA, PBS). Following hindlimb muscle excision, both femurs (*n* = 5/group) were cleaned and placed in ice cold PBS. The epiphysis of the femurs was removed, and the bone marrow was flushed with PBS using a 26G syringe. Cells were then passed through a 70-μm filter and suspended in flow buffer (2% FBS-PBS). Red blood cell lysis was performed with 20 second hypotonic solution (0.2% NaCl) treatment followed by hypertonic (1.6% NaCl) cessation. This method has been shown to reduce disturbances to cell surface markers compared to alternative RBC lysis buffers (Swamydas and Lionakis, 2013). Both skeletal muscle and bone marrow cells were blocked with Fc-block against CD16 and CD32 in their respective flow buffers. Cells were then incubated with fluorescently labeled antibodies against CD45 (PE/CY7), CD11b (APC), Ly6c (PerCP/Cy5.5), F4/80 (FITC), CD11c (APC/Cy7), and CD206 (PE). Cells were measured using a FACS Aria II and analyzed using FlowJo V10.6.2 (BD Biosciences, Ashland, OR, United States). Prior to cellular analysis, all colors were compensated using Invitrogen UltraComp eBeads^TM^ Compensation Beads (Life technologies, Carlsbad CA, United States). A total of 5 × 10^5^ skeletal muscle cells and 3 × 10^5^ bone marrow cells were analyzed.

### RNA Isolation and RT-PCR

RNA isolation, cDNA synthesis, and real-time PCR were performed as previously described (Sougiannis et al., 2019) using reagents from Applied Biosystems (Foster City, CA, United States). Briefly, RNA was extracted from the gastrocnemius using the TRIzol/isopropanol/chloroform procedure (Life Technologies, GIBCO-BRL, Carlsbad, CA, United States). RNA sample quality and quantities were verified using a Nanodrop One Microvolume UV-Vis Spectrophotometer (Thermo Fisher Scientific, Waltham, MA, United States) and determined to be of good quality based on A260/A280 values (>1.8) prior to cDNA synthesis using High capacity Reverse Transcriptase kit (Applied Biosystems, Foster City, CA, United States). Probes for MCP-1, IL-6, IL-1β, IL-10, TNF-α, IFNγ, CD11c, CD206, F4/80, and CD68 as well as housekeeping genes Hmbs, B2M, TBP, H2afv, and 18s were purchased from Applied Biosystems (Foster City, CA, United States). Quantitative RT-PCR analysis was carried out as per the manufacturer’s instructions (Applied Biosystems, Foster City, CA, United States) using Taq-Man Gene Expression Assays on a Qiagen Rotor-Gene Q. Data were normalized to vehicle treated controls and compared to five reference targets (Hmbs, B2M, TBP, H2afv, and 18s), which were evaluated for expression stability using GeNorm (St-Pierre et al., 2017).

### Statistics

Values are presented as means ± standard error of the mean (SEM). Student *t*-tests were performed to determine the differences between 5FU and Control for all endpoint measurements. A repeated measures two-way ANOVA was used to determine a difference in body weight change and food intake (treatment × time). *Post hoc* analysis were performed with student Newman-Keuls methods. A Bartlett’s test was used to determine significantly different standard deviations. Significance was set at *p* ≤ 0.05.

## Results

### Animal Characteristics

**FIGURE 1:**
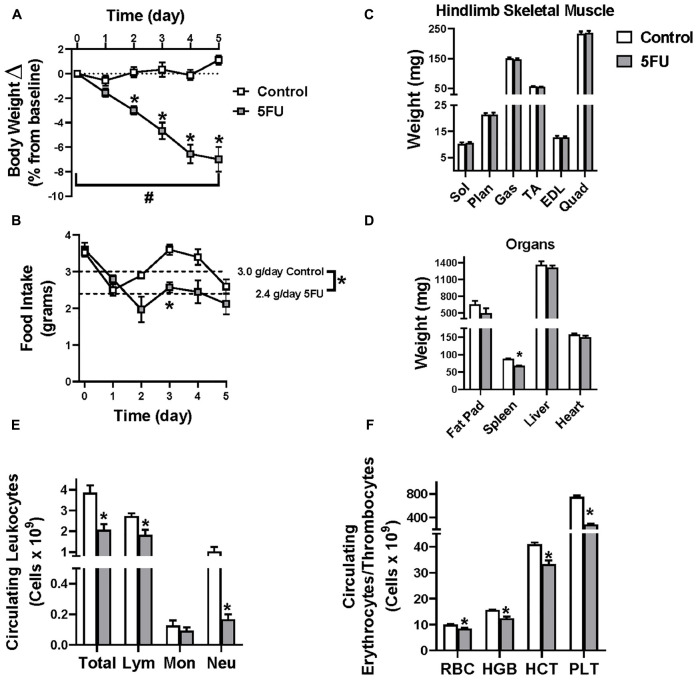
Animal characteristics. 5-fluorouracil (5FU) was solubilized in phosphate buffered saline at 3.5 mg/mL and administered to the mice at 35 mg/kg via intraperitoneal injection once daily for 5 days. **(A)** Relative body weight change shown as the % change from day 0 throughout the duration of the study. **(B)** Daily food intake in grams throughout the duration of the study. Dotted line illustrates the average daily food intake in grams (g) per day over the course of the 5 days of treatment. **(C)** Select hindlimb muscle weights given in milligrams (mg) after 5 days of 5FU. **(D)** Select organ weights in mg after 5 days of 5FU. **(E)** Circulating leukocytes given as # of cells × 10^9^/L after 5 days of 5FU. **(F)** Circulating erythrocytes and thrombocytes given as # of cells × 10^9^/L after 5 days of 5FU. Sol, soleus; Plan, plantaris; Gas, gastrocnemius; TA, tibialis anterior; EDL, extensor digitorum longus; Quad, quadriceps; Lym, lymphocytes; Mon, monocytes; Neu, neutrophils; RBC, red blood cells; HBG, hemoglobin; HCT, hematocrit; PLT, platelets. Significance was set at *p* < 0.05. *Significantly different from Control using a student’s *t*-test. #Significantly different from Day 0 using a repeated measures Two-way ANOVA.

Body weights were monitored daily during the treatment period and shown as a relative change (%) from Day 0. 5FU treated mice exhibited body weight loss between day 0 and Day 5 (effect of time; *p* < 0.0001) and had reduced % body weight between days 2 and 5 (2–8%) compared to controls (*p* < 0.0001) (Figure 1A). 5FU reduced the overall average daily food intake (g/day) by 20.5% (*p* = 0.006) compared to controls (Figure 1B). Despite the reductions in body weight and food intake, there were no observed differences between 5FU and controls in several hindlimb muscle weights, soleus (*p* = 0.35), plantaris (*p* = 0.96), gastrocnemius (*p* = 0.57), extensor digitorum longus (*p* = 0.99), tibialis anterior (*p* = 0.50), and quadriceps (*p* = 0.82) (Figure 1C). Spleen weight was decreased 22.6% (*p* < 0.0001) with 5FU (Figure 1D) which is further supported by a 46.4% (*p* = 0.001) decrease in circulating leukocytes (Figure 1E). More specifically, circulating lymphocytes and neutrophils were reduced with 5FU by 33.3% (*p* = 0.006) and 83.5% (*p* = 0.002), respectively, with no significant change in circulating monocytes (26.5% reduction, *p* = 0.43) (Figure 1E). 5FU reduced circulating red blood cells (RBC) by 16.2% (*p* = 0.002) with 20.8% (*p* = 0.0003) and 18.8% (*p* = 0.0002) reductions in hemoglobin (HGB) and hematocrit (HCT), respectively (Figure 1F). Additionally, 5FU decreased platelets (PLT) by 62.6% (*p* < 0.0001; Figure 1F).

### The Effect of 5FU on Skeletal Muscle Monocytes

**FIGURE 2:**
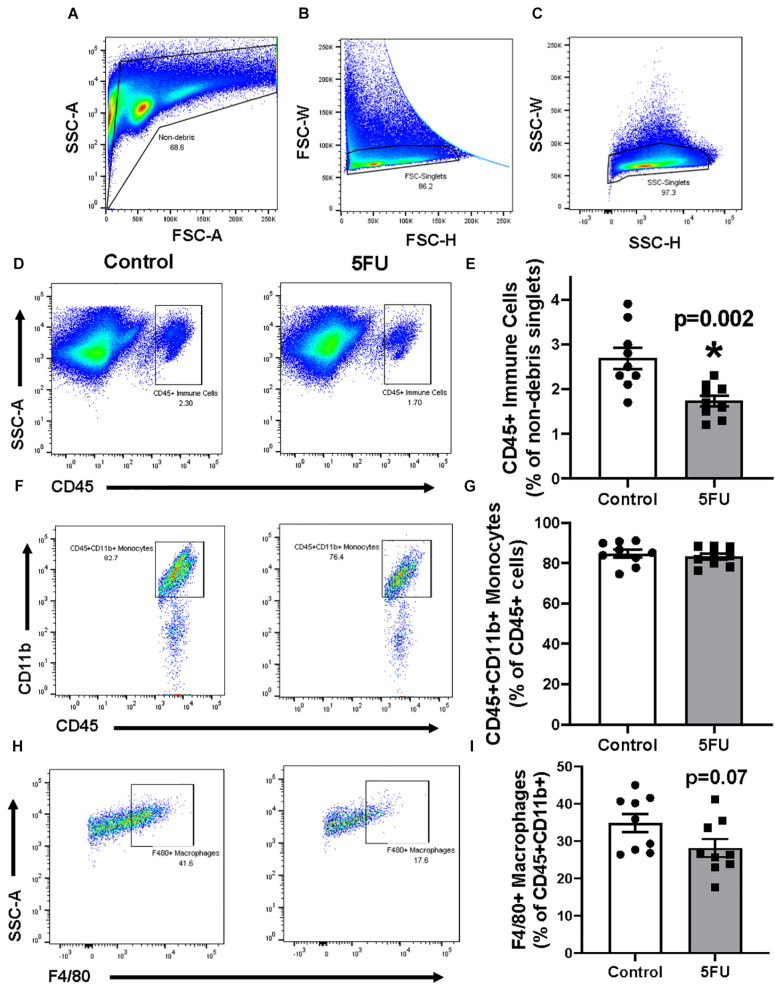
The effects of 5FU on skeletal muscle immune cells. **(A)** Cells were gated for non-debris (SSC-A × FSC-A), **(B)** FSC singlets (FSC-W × FSC-H), **(C)** and SSC singlets (SSC-W × SSC-H; A-right). **(D)** Non-debris singlet cells were then gated for total immune cells with CD45+. **(E)** CD45+ cells were quantified and shown in the bar graph as the relative % of non-debris singlets. **(F)** CD45+ cells were gated for monocytes with CD11b+. **(G)** CD45+CD11b+ cells were quantified and shown in the bar graph as the relative % of CD45+ cells. **(H)** CD45+CD11b+ were then gated for macrophages with F4/80. **(I)** F4/80+ cells were quantified and shown in the bar graph as the relative % of CD45+CD11b+ cells. Significance was set at *p* < 0.05. *Significantly different from Control using a student’s *t*-test.

Cells isolated from the quadriceps underwent the following gating procedures, which was previously described (Reidy et al., 2019). Cells were first gated for “non-debris” by plotting SSC-A × FSC-A (Figure 2A). Cells were then gated for single cells by plotting SSC-W × SSC-H (Figure 2B) and then FSC-W × FSC-H (Figure 2C). Immune cells were then gated from “non-debris,” “SSC singlets,” and “FSC singlets” by plotting SSC-A by CD45. CD45+ cells were considered all immune cells and were quantified as a % of singlets (Figure 2D) and total number of immune cells (Table 1). 5FU treatment resulted in a 35.5% decrease (*p* = 0.003) in the relative quantity of CD45+ immune cells (Figure 2E), and a 46.9% decrease in total CD45+ immune cells (Table 1). CD45+ immune cells were further gated with CD11b and CD45+CD11b+ cells were classified as monocytes and were quantified as a % of CD45+ cells (Figure 2F) and total number of monocytes (Table 1). The relative abundance of monocytes within CD45+ cells was not significantly different (1.4%, *p* = 0.57) with 5FU treatment (Figure 2G); however, total monocytes were reduced by 47.0% with 5FU (Table 1). CD45+CD11b+ cells were further gated with F4/80 and CD45+CD11b+F4/80+ cells were classified as macrophages and were quantified as a % of CD45+CD11b cells (Figure 2H) and total number of macrophages (Table 1). 5FU decreased the relative abundance of macrophages by 19.2% within CD45+CD11b+ monocytes; however, this did not reach statistical significance (*p* = 0.07; Figure 2I). 5FU reduced total macrophage count by 56.8% (Table 1). Last, there was a strong correlation between circulating leukocytes and skeletal muscle CD45+ immune cells (*R* = 0.75; *p* = 0.003), CD11b+ monocytes (*R* = 0.69; *p* = 0.002), F4/80+ macrophages (*R* = 0.67; *p* = 0.002), and Ly6c^High^ infiltrating monocytes (*R* = 0.67; *p* = 0.008) in all mice.

**TABLE 1:** Skeletal muscle immune cell population.

		Total	Non-debris	FSC Singlet	SSC Singlet	CD45+	CD45+ CD11b+	CD45+ CD11b+ F4/80+
Control	Mean	500000	357171	300554	293303	8313	6986	2509
	SEM	0	(9278)	(12211)	(12898)	(1112)	(1092)	(531)
5FU	Mean	500000	329763	265158	257197	4412*	3701*	1084*
	SEM	0	(12996)	(15057)	(15433)	(397)	(384)	(182)
*p*-value			0.207	0.180	0.171	0.005	0.009	0.013

Values are means ± SEM. Total number of cells counted. Absolute number of non-debris cells from the total number of cells. Absolute number of forward scatter (FSC) single cells from the non-debris cells. Absolute number of side scatter (SSC) single cells from the FSC single cells. Absolute number of CD45+ cells from the SSC single cells. Absolute number of CD45+CD11b+ cells from SSC single cells. Absolute number of CD45+CD11b+F4/80+ cells from SSC single cells. Significance was set at p < 0.05. *Significantly different from Control using a student’s t-test.

### Skeletal Muscle Inflammatory Gene Expression

**FIGURE 3:**
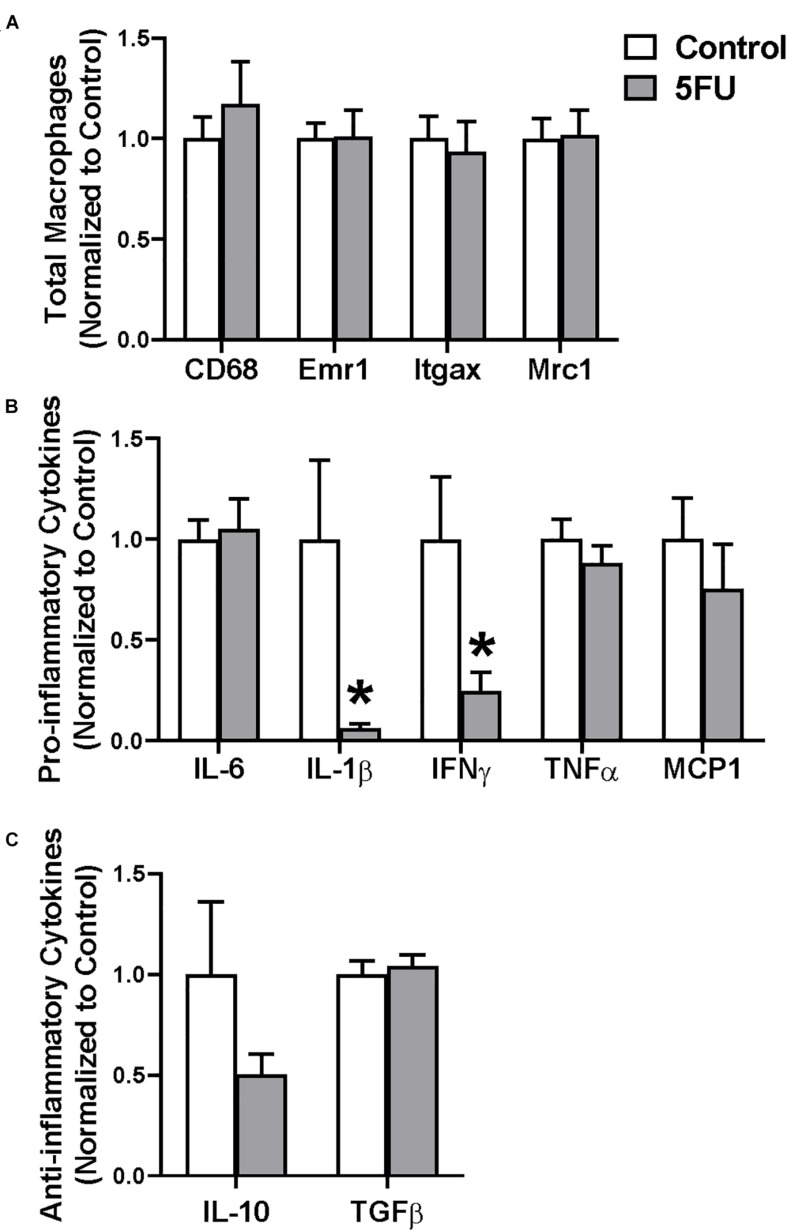
The effects of 5FU on skeletal muscle macrophage gene expression. **(A)** Relative gene expression of total macrophage genes, CD68 and Emr1 (F4/80), M1-like macrophage gene, Itgax (CD11c), and M2-like macrophage gene, Mrc1 (CD206). **(B)** Relative gene expression of pro-inflammatory genes, Interleukin (IL) 6, IL-1β, Interferon (IFN) γ, Tumor necrosis factor (TNF) α, and monocyte chemoattractant protein (MCP) 1. **(C)** Relative gene expression of anti-inflammatory genes IL-10 and transforming growth factor (TGF) β. Significance was set at *p* < 0.05. *Significantly different from Control using a student’s *t*-test.

RNA was extracted from the gastrocnemius which shares a similar myofibrillar myosin heavy chain isoform expression as the quadriceps and is similarly a prime mover. There was no difference in expression of total macrophage markers CD68 or Emr1 (F4/80) with 5FU treatment (Figure 3A). Additionally, there was no difference in M1-like macrophage gene Itgax (CD11c) or M2-like macrophage gene Mrc1 (CD206) with 5FU (Figure 3A). 5FU reduced pro-inflammatory cytokines, IL-1β and IFNγ, 95% (*p* = 0.009) and 75% (*p* = 0.01), respectively, while IL-6, TNFα, and MCP-1 were not changed (Figure 3B). There were no differences in anti-inflammatory cytokines IL-10 and TGFβ with 5FU (Figure 3C).

### The Effect of 5FU on Resident and Infiltrating Skeletal Muscle Monocytes and Macrophages

**FIGURE 4:**
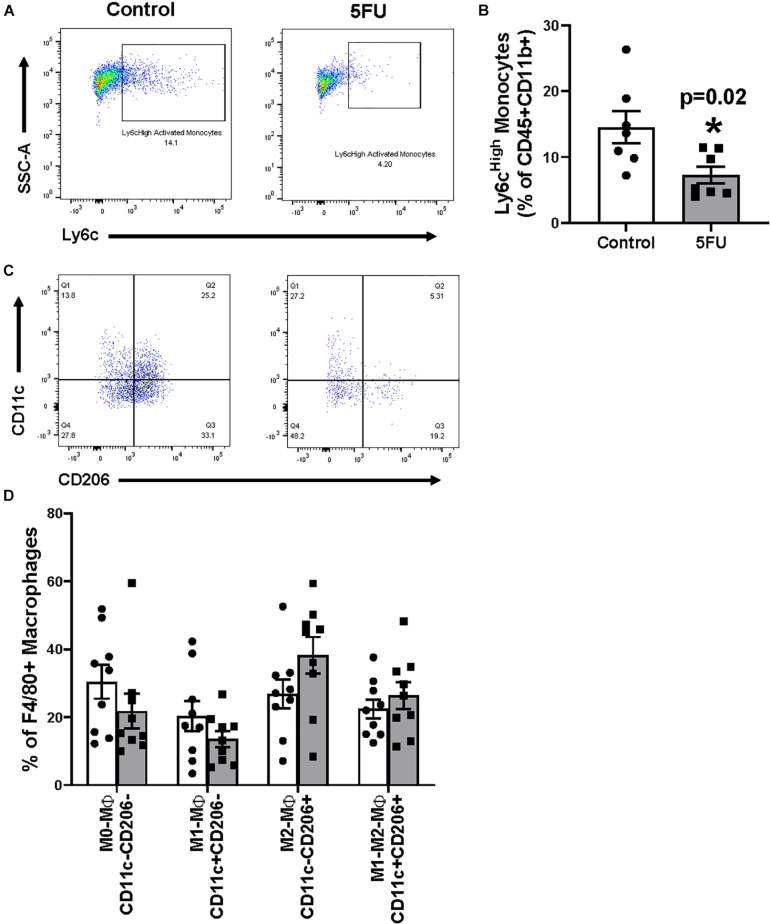
The effects of 5FU on infiltrating skeletal muscle monocytes and macrophages. **(A)** CD11b+ monocytes were gated for their activation status using Ly6C. Cells were considered either resident (Ly6cLow) or activated/infiltrating (Ly6cHigh). **(B)** Ly6cHigh monocytes were quantified and shown in the bar graph as relative % of CD45+CD11b+ cells. **(C)** F4/80+ macrophages were gated analyzed for their polarization status using CD11c and CD206. **(D)** CD11c-CD206- cells were considered M0-like macrophages, CD11c+CD206- cells were considered M1-like macrophages, CD11c-CD206+ cells were considered M2-like macrophages, and CD11c+CD206+ cells were considered M1-M2-like transitional macrophages and graphed as the relative % of F480+ macrophages. Significance was set at *p* < 0.05.*Significantly different from control (*t*-test).

Given the decrease in pro-inflammatory cytokines IL-1β and IFNγ, we sought to understand the phenotype of skeletal muscle monocytes. Similar to Figure 2, cell singlets were gated for CD45+CD11b+ followed by Ly6C to understand the effects of 5FU on infiltrating monocytes. Ly6c^High^ cells were classified as infiltrating monocytes and were quantified as a % of CD45+CD11b+ cells (Figure 4A) and total number of activated monocytes (Table 2). 5FU decreased the relative abundance of Ly6c^High^ infiltrating monocytes by 49.9% (*p* = 0.02) within CD45+CD11b+ monocytes (Figure 4B). Also, total Ly6c^High^ infiltrating monocytes were reduced by 73.0% with 5FU, but this did not achieve statistical significance (*p* = 0.06; Table 2). Total Ly6c^Low^, resident monocytes, were reduced by 38.6% with 5FU (Table 2). Given that the total number of macrophages were reduced with 5FU (Table 1), we examined if there were changes in macrophage phenotype by measuring CD11c (M1-like) and CD206 (M2-like) from parent CD45+CD11b+F4/80+ macrophages (Figure 4C). There were no observed changes in the relative abundance of M1-like (*p* = 0.20), M2-like (*p* = 0.12), M1-M2 transitional (*p* = 0.42), or M0 (*p* = 0.24) macrophages with 5FU treatment (Figure 4D); however, 5FU decreased total number of M1-like (CD11c+CD206-) by 70.7%, M1-M2 transition (CD11c+CD206+) by 63.6%, and M0 (CD11c-CD206-) macrophages by 57.0% (Table 2). The total number of M2-like macrophages (CD11c-CD206+) cells were not significantly different between groups (Table 2).

**TABLE 2:** Skeletal muscle activated monocyte population.

		CD45+ CD11b+									
								F4/80+			
		Ly6c^High^	Ly6c^Low^	F480- Ly6c^High^	F480+ Ly6c^High^	F480+ Ly6c^Low^	F480- Ly6c^Low^	CD206- CD11c+	CD206+ CD11c+	CD206+ CD11c-	CD206- CD11c-
Control	Mean	1161	5824	685	475	1806	4018	852	385	646	625
	SEM	(398)	(701)	(230)	(168)	(337)	(387)	(222)	(131)	(150)	(132)
5FU	Mean	313*	3576*	119*	193	842*	2733*	250*	140*	423	269*
	SEM	(87)	(389)	(28)	(59)	(144)	(276)	(86)	(25)	(100)	(48)
*p*-value		0.060	0.016	0.031	0.141	0.022	0.019	0.030	0.015	0.248	0.044

Values are means ± SEM. Absolute number of Ly6c^High^ cells from the CD45+CD11b+ single cells. Absolute number of Ly6c^Low^ cells from the CD45+CD11b+ single cells. Absolute number of F4/80-Ly6c^High^ cells from the CD45+CD11b+ single cells. Absolute number of F4/80+Ly6c^High^ cells from the CD45+CD11b+ single cells. Absolute number of F4/80+Ly6c^Low^ cells from the CD45+CD11b+ single cells. Absolute number of F4/80-Ly6c^Low^ cells from the CD45+CD11b+ single cells. Absolute number of CD206-CD11c+ cells from the CD45+CD11b+F4/80+ single cells. Absolute number of CD206+CD11c+ cells from the CD45+CD11b+F4/80+ single cells. Absolute number of CD206+CD11c- cells from the CD45+CD11b+F4/80+ single cells. Absolute number of CD206-CD11c- cells from the CD45+CD11b+F4/80+ single cells. Significance was set at p < 0.05. *Significantly different from Control using a student’s t-test.

### The Effects of 5FU on the Bone Marrow

**FIGURE 5:**
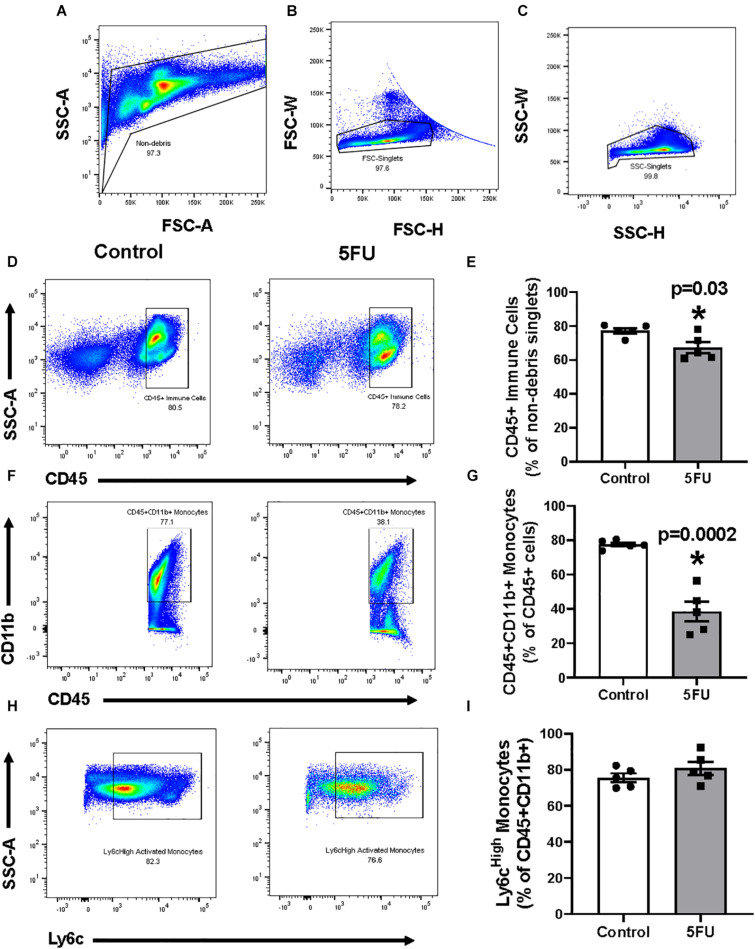
The effects of 5FU on bone marrow immune cells. **(A)** Cells were gated for non-debris (SSC-A × FSC-A), **(B)** FSC singlets (FSC-W × FSC-H), **(C)** and SSC singlets (SSC-W × SSC-H; A-right). **(D)** Non-debris singlet cells were then gated for total immune cells with CD45+. **(E)** CD45+ cells were quantified and shown in the bar graph as the relative % of non-debris singlets. **(F)** CD45+ cells were gated for monocytes with CD11b+. **(G)** CD45+CD11b+ cells were quantified and shown in the bar graph as the relative % of CD45+ cells. **(H)** CD11b+ monocytes were gated for their activation status using Ly6C. Cells were considered either resident (Ly6cLow) or activated/infiltrating (Ly6cHigh). **(I)** Ly6cHigh monocytes were quantified and shown in the bar graph as relative % of CD45+CD11b+ cells. Significance was set at *p* < 0.05. *Significantly different from Control using a student’s *t*-test.

**FIGURE 6:**
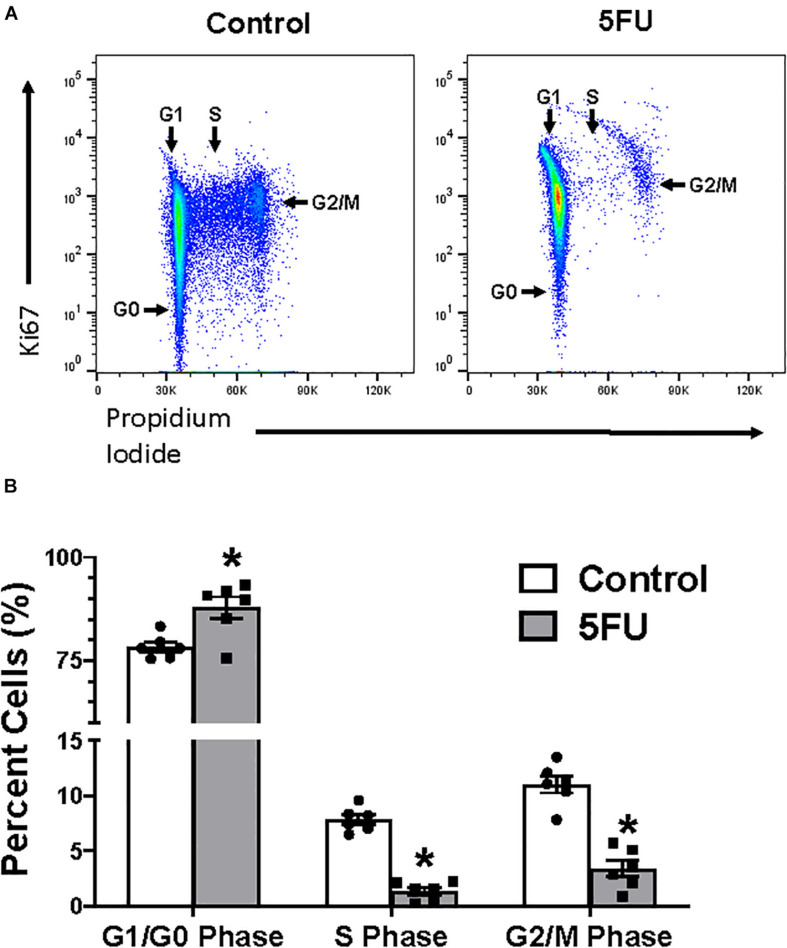
The effects of 5FU on bone marrow cell cycle. **(A)** Cells were fixed and stained with Ki67 and propidium iodide (PI). **(B)** Cells in the G1/G0, S, and G2/M phases were quantified and shown in the bar graph as the relative % of total cells. Significance was set at *p* < 0.05.*Significantly different from control (*t*-test).

In order to further understand the effects of 5FU on circulating and infiltrating monocytes, we examined 5FU’s impact on bone marrow cells (Figure 5). Bone marrow isolates were obtained from both femurs of 5 mice/group. Cell gating of bone marrow cells was performed as described for data in Figures 2, 5A–D. 5FU decreased the relative abundance of CD45+ immune cells by 12.9% (*p* = 0.03; Figure 5E) and total CD45+ immune cells by 19.3% (Table 3), but the reduction in total CD45+ immune cells did not reach statistical significance (*p* = 0.096). CD45+ cells were further gated with CD11b and CD45+CD11b+ cells were considered monocytes (Figure 5F) and were quantified as a % of CD45+ cells (Figure 5G) and total number of monocytes (Table 3). 5FU treatment reduced the relative abundance of bone marrow monocytes by 50.3% (*p* = 0.0002) within total CD45+ immune cells (Figure 5G) and reduced total monocytes by 60.7%. CD45+CD11b+ cells were further gated with Ly6C and CD45+CD11b+Ly6C^High^ cells were considered activated monocytes (Figure 5H) and were quantified as a % of CD45+CD11b+ cells (Figure 5G) and total number of activated monocytes (Table 3). 5FU treatment had no apparent effect on the relative abundance of bone marrow activated monocytes within total CD45+CD11b+ monocytes (Figure 5I); however, the total number of activated monocytes was reduced by 53.2% (Table 3). Additionally, 5FU induced cell cycle arrest in the bone marrow (Figure 6). 5FU increased the relative abundance of cells in the G1/G0 cell cycle phase by 12.0% (*p* = 0.009) and decreased S and G2/M cell cycle phases by 82.4% (*p* < 0.0001) and 69.1% (*p* < 0.0001), respectively (Figure 6B).

**TABLE 3:** Bone marrow immune cell population.

		Total	Non-debris	FSC Singlet	SSC Singlet	CD45+	CD45+ CD11b+	CD45+ CD11b+ Ly6c^High^
Control	Mean	500000	483227	465831	464058	304878	233494	160170
	SEM	0	(1160)	(2145)	(2295)	(50575)	(44979)	(40441)
5FU	Mean	500000	409499*	401998*	400736*	246100	91678*	74938*
	SEM	0	(8611)	(8845)	(8948)	(26514)	(19190)	(16942)
*p*-value			0.00001	0.00003	0.00003	0.096	0.001	0.010

Values are means ± SEM. Total number of cells counted. Absolute number of non-debris cells from the total number of cells. Absolute number of forward scatter (FSC) single cells from the non-debris cells. Absolute number of side scatter (SSC) single cells from the FSC single cells. Absolute number of CD45+ cells from the SSC single cells. Absolute number of CD45+CD11b+ cells from SSC single cells. Absolute number of CD45+CD11b+Ly6c^High^ cells from SSC single cells. Significance was set at p < 0.05. *Significantly different from Control using a student’s t-test.

## Discussion

5 fluorouracil has been the first-choice chemotherapy drug for several cancer types; however, its efficacy is diminished by patient acquired resistance and pervasive side effects contributing to reduced life quality and poor treatment outcomes (van Kuilenburg and Maring, 2013; Giuliani and Bonetti, 2016; Lee et al., 2016; McQuade et al., 2017). Given 5FU’s deleterious effects on circulating leukocytes, the purpose of our study was to investigate the acute effects of 5FU on resident and infiltrating skeletal muscle monocytes and inflammatory mediators. Our results extend previous studies to identify that 1 cycle of a clinically translatable dose of 5FU significantly reduced CD45+ immune cells and infiltrating/activated CD11b+Ly6C^High^ monocytes in skeletal muscle that was associated with a decrease in select skeletal muscle inflammatory mediators. Additionally, the reduction in skeletal muscle was accompanied by a reduction in bone marrow monocytes and an increase in cell cycle arrest. These results identify novel off-target effects of 5FU on skeletal muscle and the skeletal muscle microenvironment independent of muscle mass regulation.

Our understanding of chemotherapy-induced body weight and function loss, termed cachexia, has improved over the last decade (Gilliam et al., 2009; Barreto et al., 2016b; Morton et al., 2019). Our investigation of the acute (1 cycle) effects of 5FU demonstrated that 5FU induced clinically relevant body weight loss (>5%) (Evans et al., 2008), which was accompanied with signs of anorexia, but not skeletal muscle mass loss. Others have demonstrated that 5 weeks of 5FU combination therapy, Folfiri (leucovorin, 5FU, Irinotecan), decreased body weight and lean mass over time, with corresponding reductions in several hindlimb weights (Barreto et al., 2016b). Interestingly, mice given 5 weeks of Folfox (leucovorin, 5FU, oxaliplatin) rather than Folfiri maintained body weight and lean mass, and only showed reduced quadriceps weight. Additionally, similar to the doxorubicin effects on skeletal muscle (Gilliam et al., 2009; Tarpey et al., 2019), only Folfiri reduced skeletal muscle specific force (strength per muscle unit area) – which may occur through several mechanisms including fibrosis (Barreto et al., 2016b). Together this suggests that significant muscle mass loss only occurs after sustained 5FU treatment (Barreto et al., 2016b, 2017), given that 1 week of 5FU was unable to reduce hindlimb muscle weight. We then hypothesize that it is likely that anorexia and dehydration contribute to the observed body weight loss as these are commonly reported with 5FU (Liaw et al., 1999; Yi et al., 2016). Additionally, the potential for body weight loss and anorexia alone to contribute to the observed immune disruptions cannot be ruled out. Unfortunately, however, a weight loss only group was not included to test this hypothesis therefore limiting our interpretations. Nonetheless, our results provide evidence to suggest that 5FU-induced anemia and leukopenia are likely to contribute to the observed functional pathologies that occur with 5FU treatment.

The role of immune cells, particularly macrophages, in skeletal muscle regeneration, repair, and remodeling has been well characterized (Tidball, 2017); however, chemotherapy’s effects on these processes is not well known. Following skeletal muscle insult (e.g., damage, ischemia, exercise), there is an initial influx of neutrophils which in turn recruit naïve monocytes primarily through the release of MCP-1 (Deshmane et al., 2009; Tidball, 2017). We have previously shown that 5FU induced circulating MCP-1 after 14 days of treatment, which was associated with reduced voluntary physical activity (Mahoney et al., 2013). The monocytes recruited by MCP-1 are primarily recruited as CD11b+Ly6C^High^ monocytes which can either remain as such or differentiate and polarize to a pro-inflammatory M1-like F4/80+CD11c+CD206- macrophage (Tidball, 2005; Guilliams et al., 2014; Yang et al., 2014). Following an acute 5FU regime (1 week) we document a reduction in total and relative Ly6C^High^ monocytes as well as total M1-like F4/80+CD11c+CD206- macrophages in skeletal muscle despite no changes in skeletal muscle pro-inflammatory MCP-1, IL-6, and TNFα levels. However, we did observe decreased expression of pro-inflammatory genes associated with M1-like macrophages, IL-1β and IFNγ, but on the other hand did not observe corresponding changes to total M1-like macrophage cell surface marker, Itgax, more commonly known as CD11c (Jablonski et al., 2015). These discrepancies between the flow cytometry and gene transcription require additional work and thus, interpretations should be taken with caution; however, flow cytometry remains the gold standard for the assessment of immune cells, and it appears evident that 5FU has deleterious effects on the pro-inflammatory monocytes and macrophages. A loss of pro-inflammatory or phagocytic M1-like macrophages could negatively impact skeletal muscle remodeling and repair (Tidball, 2005). Chemotherapeutic doxorubicin has been shown to blunt the pro-inflammatory response following exercise which mitigated the muscle’s response to exercise (Huang et al., 2017). Furthermore, while repeated muscular contractions were able to improve muscle mass in cancer patients undergoing treatment, patients did not obtain the functional and metabolic improvements that have been previously seen with exercise (Guigni et al., 2019; Toth et al., 2020). While chemotherapeutics 5FU and doxorubicin mechanisms of action differ, we can still glean potential mechanisms and clinical manifestations. To the best of our knowledge, we are the first to document that 5FU disrupts skeletal muscle’s pro-inflammatory immune cell environment. It is also important to note that these cell surface markers and the M1/M2 dichotomous classification of macrophages does not properly reflect the true diversity and nature of resident/infiltrating macrophages and should again be interpreted cautiously (Davies et al., 2013; Guilliams et al., 2014; Martinez and Gordon, 2014; Tidball, 2017).

Tissue resident macrophages are classically CD206+ anti-inflammatory, pro-fibrotic surveying macrophages (Gordon and Taylor, 2005; Murray and Wynn, 2011; Cote et al., 2013; Guilliams et al., 2014); however, macrophages are plastic and as skeletal muscle repair progresses the infiltrated M1-like F4/80+CD11c+ macrophages can reduce the gene expression and release of pro-inflammatory mediators and become more phenotypically M2-like to promote extracellular matrix remodeling and angiogenesis (Schiaffino et al., 2017; De Santa et al., 2018; Shapouri-Moghaddam et al., 2018). Others have proposed that resident macrophages are predominantly M0 (CD11c-CD206-) which are self-maintained, proliferate, and polarize to an M1-like phenotype upon activation during the initial stages of injury repair (Cote et al., 2013; Tidball, 2017). Regardless, our results demonstrate that the relative phenotype of skeletal muscle macrophages is not changed by 5FU treatment; however, the total number of M1-like (CD11c+CD206-), M0-like (CD11c-CD206-), and M1-M2-like transitional macrophages were reduced with 5FU while M2-like macrophages appear spared from 5FU’s cytotoxicity – at least following 1 week of 5FU. Additionally, anti-inflammatory IL-10, pro-fibrotic TGFβ, and M2-like macrophage cell surface marker Mrc1, commonly known as CD206, gene transcription were not changed by 5FU treatment. The potential for 5FU to target M1-like macrophages rather than M2-like, points to a pro-fibrotic skeletal muscle microenvironment. 5FU combination therapy Folfiri was shown to reduce skeletal muscle specific force (force per unit area); however, neither fibrosis nor an increase in fibrotic genes (TGF-β associated ligands) were apparent (Barreto et al., 2016b). Therefore, it is likely that these pro-fibrotic M2-like cells remain at a physiological abundance during 5FU treatment and may not be contributing to a skeletal muscle pathology directly. Interestingly, TAMs phenotypically reflect M2-like macrophages promoting immunosuppression, fibrosis, and angiogenesis, within the tumor microenvironment and have been associated with 5FU acquired resistance (Zhang et al., 2016). The potential for M2-like macrophages to be protected against 5FU requires significant attention in the cancer domain.

Chemotherapy has been shown to mitigate the inflammatory response with exercise (Huang et al., 2017; Smuder, 2019), induce leukopenia/cytopenia (Shitara et al., 2011), and disrupt cardiac macrophage infiltration (Johnson and Singla, 2018). To the best of our knowledge, this is the first study to demonstrate that chemotherapeutic 5FU has deleterious effects on immune cell abundance in otherwise healthy uninjured skeletal muscle. The absolute reduction in macrophage number rather than relative changes in abundance remains relevant given the physiological importance of the overall immune response in repair and remodeling (Summan et al., 2006; Segawa et al., 2008; Cote et al., 2013; Xiao et al., 2016; Zhao et al., 2016; Inaba et al., 2018). The mean age of cancer patients is ∼65 years and overlapping sarcopenic and cachectic factors along with chemotherapy may contribute to disrupted skeletal muscle immune regulation (Dunne et al., 2019). Disrupted skeletal muscle repair associated with changes in macrophages has been reported with aging (Reidy et al., 2019), cancer (Inaba et al., 2018), and chemotherapy (Huang et al., 2017). The effects of aging on skeletal muscle macrophages has demonstrated that reloading aged skeletal muscle had a blunted hypertrophy response associated with a lower number of M1-like macrophages at baseline and blunted M1-like macrophage infiltration (early) and M2-like macrophage transition (late) (Reidy et al., 2019). Surprisingly, while inflammation is a hallmark of cancer cachexia associated with muscle weakness and fatigue (VanderVeen et al., 2017, 2018), total macrophage number was reduced in damaged muscle of C26 tumor-bearing mice compared to a non-cachectic tumor-bearing control (Inaba et al., 2018). Additionally, macrophages were shown to regulate skeletal muscle signal transducer and activator of transcription 3 (STAT3) – downstream target of IL-6 and key regulator of skeletal muscle mitochondrial homeostasis and proteostasis (Carson and Kristen, 2010; Bonetto et al., 2011, 2012; VanderVeen et al., 2017, 2019) – during pancreatic cancer cachexia (Shukla et al., 2020). Further work is needed to understand these potentially overlapping mechanisms with cancer and chemotherapy on skeletal muscle immune cells.

Chemotherapy’s effects on systemic inflammatory mediators (Lee et al., 2014; Ribeiro et al., 2016; Derman et al., 2017; Sougiannis et al., 2019) and intrinsic skeletal muscle inflammatory signaling (Kvinnsland, 1999; Yamanaka et al., 2007; Shitara et al., 2009; Baechler et al., 2010; Han et al., 2012; Abraham et al., 2015) are continuing to be unearthed; however, our study is the first to identify that 5FU-induced leukopenia extends beyond circulation to impact the skeletal muscle microenvironment. Our results indicate that 5FU’s toxic effects on skeletal muscle leukocytes are not necessarily specific to monocytes shown by no change in both circulating monocyte count or relative abundance of skeletal muscle CD11b+ monocytes within the CD45+ population. This is not to say that monocytes are spared from 5FU as the total number of skeletal muscle monocytes are reduced. Bone marrow CD11b+ monocytes and the relative abundance of infiltrating Ly6C^High^ monocytes in skeletal muscle were reduced with 5FU which is supported by the established deleterious effects of 5FU on circulating leukocytes and the hematopoietic system (Yamanaka et al., 2007; Lee et al., 2016; Sougiannis et al., 2019). In conjunction with previous studies, our results support that 5FU’s toxicity is predominantly associated with pro-inflammatory mediators extending beyond the hematopoietic system to impact the skeletal muscle microenvironment. Another potential mechanism for the observed impact of 5FU on skeletal muscle immune cells is the potential for reduced proliferation of pro-inflammatory macrophages within the muscle microenvironment. Given that circulating and skeletal muscle monocytes/macrophages are not proportionally reduced it is possible that 5FU increased maturation of monocytes within the skeletal muscle as well as increased proliferation of M2-like macrophages.

## Conclusion

Understanding chemotherapy’s off-target effects will allow for improvements to treatment efficacy aimed at increasing cancer patient survival and quality of life. Our novel finding that chemotherapeutic 5FU depletes skeletal muscle immune cells and infiltrating monocytes provides insight into the skeletal muscle microenvironment that may contribute to weakness, fatigue, and treatment intolerance (Williams et al., 2018). We provide evidence to suggest that 5FU reduced circulating and skeletal muscle leukocytes through disrupting the hematopoietic system by inducing cell cycle arrest in the bone marrow. While our results are limited to 5FU’s acute toxicities, future studies are needed to understand the long-term implications of this loss of immune cells and if chronic 5FU exposure exacerbates this immune dysregulation. Furthermore, additional work is needed to determine if mitigating the loss of immune cells can improve skeletal muscle function following repeated cycles of 5FU.

## Data Availability Statement

The original contributions presented in the study are included in the article/supplementary material, further inquiries can be directed to the corresponding author/s.

## Ethics Statement

The animal study was reviewed and approved by the University of South Carolina’s Institutional Animal Care and Use Committee.

## Author Contributions

BV, JC, KV, DF, and EM conceived and designed the experiments. BV and AS performed the experiments. BV prepared the figures and drafted the manuscript. All authors edited, revised, and approved final version of the manuscript.

## Conflict of Interest

BV, DF, and EM were employed by the company AcePre LLC. The remaining authors declare that the research was conducted in the absence of any commercial or financial relationships that could be construed as a potential conflict of interest.

## Abbreviations

5FU: 5 fluorouracil
BSA: bovine serum albumin
CD: cluster of differentiation
DMEM: Dulbecco’s Modified Eagle Medium
EDTA: Ethylenediaminetetraacetic acid
FBS: fetal bovine serum
FSC: forward scatter
HCT: Hematocrit
HGB: Hemoglobin
IFN: interferon
IL: Interleukin
Ly6c: lymphocyte antigen 6c
LYM: lymphocyte
MCP: monocyte chemoattractant protein
MON: monocyte
NEU: neutrophil
PBS: phosphate buffered saline
PLT: platelets
RBC: red blood cell
SEM: standard error of the mean
SSC: side scatter
STAT: signal transducer and activator of transcription
TAM: tumor associated macrophage
TGF: transforming growth factor
TNF: tumor necrosis factor
WBC: white blood cell.
